# Prognostic potential of inflammatory markers in chronic kidney disease patients combined with acute myocardial infarction

**DOI:** 10.3389/fcvm.2024.1430215

**Published:** 2024-12-19

**Authors:** Peizhu Dang, Bohan Li, Yongxin Li

**Affiliations:** ^1^ Department of Cardiovascular Medicine, The First Affiliated Hospital of Xi'an Jiaotong University, Xi’an, China; ^2^ Department of Cardiovascular Surgery, The First Affiliated Hospital of Xi’an Jiaotong University, Xi’an, China

**Keywords:** acute myocardial infarction, chronic kidney disease, inflammatory markers, neutrophil-to-lymphocyte ratio, prognosis

## Abstract

**Background:**

Inflammation significantly impacts chronic kidney disease (CKD) and acute myocardial infarction (AMI). This study investigates the prognostic value of inflammatory markers in predicting outcomes for CKD patients with AMI.

**Methods:**

We enrolled patients diagnosed with CKD concomitant with AMI, choosing five inflammatory markers related to both diseases. Patients were categorized into elevated inflammatory markers group and control group based on inflammatory markers cut-off values for predicting in-hospital major adverse cardiac and cerebrovascular events (MACCE). Using univariate and multivariate logistic regression, we identified inflammation-related risk factors for MACCE. We adjusted covariates stepwise to explore the relationship between independent risk factors and adverse outcomes. We also evaluated the predictive value of these markers for MACCE by receiver operating characteristic (ROC) curves.

**Results:**

In the multivariate logistic regression analysis, higher levels of neutrophil-to-lymphocyte ratio (NLR), and platelet to lymphocyte ratio (PLR) significantly increased risk of MACCE (all *P* < 0.05). After adjusting above two indicators, NLR was independently associated with in-hospital MACCE in CKD patients with AMI (OR = 10.764, 95% CI: 1.887–61.406, *P* = 0.007). Furthermore, compared to other inflammatory markers, NLR had the highest predictive value for MACCE in patients with AMI and CKD [Area Under the Curve (AUC): 0.748, 95% Confidence Interval (CI): 0.634–0.861, *P* < 0.001].

**Conclusion:**

In CKD patients combined with AMI, elevated levels of inflammation markers could increase the risk of MACCE. NLR may provide superior predictive value compared to other markers.

## 1 Introduction

Chronic kidney disease (CKD) is caused by diverse pathological pathways irreversibly impacting kidney function and structure. According to the World Health Organization's global health statistics, 1.5% people died from CKD of global deaths (1). Persistent low-grade inflammation is considered as a key component of CKD, contributing to its pathophysiology and increasing cardiovascular and all-cause mortality (2). Acute myocardial infarction (AMI), triggered by disruptions in atherosclerotic plaques, continues to affect over 7 million annually despite declining death rates due to improved treatments (3). Previous studies have confirmed the significant pathophysiological importance of pro-inflammatory pathways in coronary artery disease (4).

Current guidelines clearly categorize patients with CKD as a high-risk group for cardiovascular diseases (CVD) and other adverse prognoses. CKD is associated with increased all-cause and cardiovascular mortality, which may be attributed to the frequent co-occurrence of multiple cardiovascular risk factors in CKD patients, including dyslipidemia, elevated inflammatory markers, hypertension, smoking, and diabetes (5). In a large national database, the prevalence of myocardial infarction among CKD patients is 10%, approximately 2–3 times higher than that in non-CKD patients. Furthermore, following AMI, CKD is associated with elevated short-term and long-term mortality rates (6).

Recent research highlights the critical role that inflammation plays in the development of various diseases, including myocardial infarction, stroke, renal failure, diabetes, and pulmonary diseases (7). The inflammatory response is not only a local process but can also have systemic manifestations, as evidenced by the increase in inflammatory markers in patients with CKD, including acute-phase proteins, cytokines, and adhesion molecules. Clinically, assessing these markers is a useful tool for predicting disease progression and prognosis (8). Previous research has found that both acute and chronic pro-inflammatory states exist in patients with CKD and that inflammation leads to increased morbidity and mortality (9). In patients with CKD, traditional mediators of chronic inflammation interact with vascular endothelial cells, causing vascular plaque formation and arteriosclerosis (10–12). Peter Stenvinkel et al. have confirmed that pro-inflammatory factors promote the development of atherosclerotic cardiovascular diseases in CKD patients through multiple pathological pathways (13). However, researches on the predictive value of inflammatory markers for adverse outcomes in CKD patients with AMI remain insufficient.

Therefore, it is important to explore inflammation biomarkers of prognosis in CKD patients combined with AMI. This study aims to initiate a single-center study, retrospectively enrolled CKD patients combined with AMI from the First Affiliated Hospital of Xi'an Jiaotong University and analyzed the impact of inflammation biomarkers on short-term prognosis in these patients. This research tends to improve survival quality, reduce the occurrence of adverse outcomes, provide a basis for enhancing clinical management of these patients.

## 2 Methods

### 2.1 Population and groups

This cohort study enrolled patients diagnosed with CKD combined with AMI at the First Affiliated Hospital of Xi'an Jiaotong University from January 2018 to January 2023. The study followed the principles of the Declaration of Helsinki and was approved by the Ethics Committee of the First Affiliated Hospital of Xi'an Jiaotong University (No: XJTU1AF2024LSYY-119).

The criteria for inclusion were as follows: (1) patients diagnosed with CKD; (2) patients diagnosed with AMI. CKD is diagnosed based on the National Kidney Foundation's Kidney Disease Outcomes Quality Initiative (KDOQI) criteria, including a sustained estimated glomerular filtration rate (eGFR) <60 ml/(min·1.73 m^2^), albuminuria [urine albumin-to-creatinine ratio (UACR) ≥30 mg/g], or the presence of other markers of kidney damage for at least 3 months (14, 15). The diagnosis of AMI met the American College of Cardiology (ACC) and American Heart Association (AHA) criteria, which consist of: (i) chest pain persisting for more than 30 min; (ii) dynamic changes on electrocardiography; (iii) alterations in serum biomarkers indicative of myocardial injury; and (iv) coronary angiography demonstrating anatomical findings suitable for percutaneous coronary intervention (PCI) to evaluate the infarct-related artery. The clinical diagnosis of AMI was determined by the attending physician's final assessment, as documented in the discharge summary.

**Figure 1:**
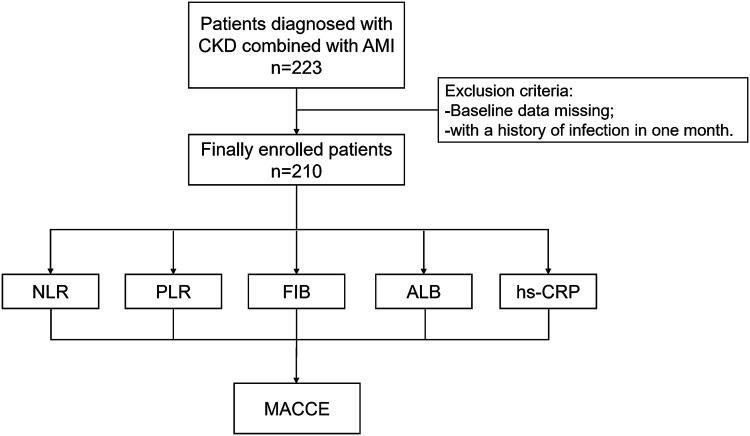
Study protocol. The flowchart of enrollment of study patients. CKD, chronic kidney disease; AMI, acute myocardial infarction; NLR, neutrophil to lymphocyte ratio; PLR, platelet to lymphocyte ratio; FIB, fibrinogen; ALB, albumin; hs-CRP, high-sensitivity C-reactive protein; MACCE, major adverse cardiac and cerebrovascular events.

The criteria for exclusion were as follows: (1) incomplete clinical data; (2) with a history of infection in one month (Figure 1).

### 2.2 Data collection

We collected demographic characteristics (age, sex), smoking history, type of AMI, comorbidities (hypertension, diabetes, valvular disease), cardiac function, coronary angiography results, and laboratory data. The laboratory data included complete blood count, liver and kidney functions, glycated hemoglobin, coagulation indicators. The inflammation markers we collected at admission were as following: neutrophil to lymphocyte ratio (NLR), platelet to lymphocyte ratio (PLR), fibrinogen (FIB), albumin (ALB), and high-sensitivity C-reactive protein (hs-CRP). We converted each continuous variable of inflammatory markers into categorical variables based on the cut-off values of each inflammatory marker in predicting endpoint events.

The primary outcome of this study was in-hospital major adverse cardiac and cerebrovascular events (MACCE), that includes in-hospital mortality, acute heart failure or acute exacerbation of chronic heart failure, malignant arrhythmias, major bleeding, and acute cerebrovascular events.

### 2.3 Measuring inflammation indicators based on blood cell counts

Blood cell counts were collected from patients at their admission to the hospital, including the total counts of white blood cells, neutrophil, lymphocyte, monocyte and platelet. Subsequently, composite inflammation ratios were calculated, including NLR and PLR. The calculation of NLR is neutrophil count/lymphocyte count. The calculation of PLR is platelet count/lymphocyte count. These inflammation biomarkers were chosen based on their potential role in AMI patients combined with CKD.

### 2.4 Statistical analysis

Continuous variables were presented as quartiles for data with a normal distribution and as mean ± standard deviation for data with a non-normal distribution. Categorical variables were expressed as percentages. Univariate logistic regression analysis was used to assess the correlation between inflammatory biomarkers and MACCE. In addition, we explored the correlation between inflammatory markers and other key biomarkers, including BNP and troponin, to investigate whether these markers are interrelated. Further adjustments were made for confounding factors to determine if each inflammatory marker was independently associated with composite outcomes after adjusting for traditional risk factors. Ultimately, all significant inflammatory markers will be included in a multivariate logistic regression analysis to identify independent risk factors associated with composite outcome events.

We conducted stepwise adjustments for independent risk factors, and performed three regression models: Model 1 was unadjusted; Model 2 adjusted for age and sex; Model 3 further adjusted for Model 2, comorbidities (hypertension, diabetes, valvular pathologies), type of AMI, ejection fraction (EF), smoking history, coronary situation and laboratory indicators. Based on the fundings from the regression analysis, a nomogram was constructed to predict the probability of composite events. The statistical analyses were performed by SPSS 27.0 (IBM Corp., NY) and R 3.1.2 (The R Foundation for Statistical Computing, Vienna, Austria). All analyses were two-sided and *P* < 0.05 was considered statistically significant.

## 3 Results

### 3.1 Clinical characteristics of study participants

A total of 210 CKD patients combined with AMI were finally enrolled, the baseline characteristics of all enrolled patients were shown in Table 1. All patients underwent coronary angiography (CAG), with 178 patients receiving PCI. A total of 36 patients had left main coronary artery disease, 155 patients had triple-vessel disease. And 70 patients had valvular pathologies. The average age of the study participants was 66 years, with 77% being male and 84% having hypertension. Additionally, 58% had diabetes, 36% had ST-segment elevation myocardial infarction, and 36% were current smokers. The average baseline eGFR was 42.9 ml/min/1.73 m^2^ and the EF was 52%.

**Table 1:** Baseline characteristics of study participants.

Variable	Study cohort
Inflammatory biomarkers	
NLR	5.1 (3.4,8.8)
PLR	5.1 (3.4,8.8)
FIB (g/L)	4.2 (3.3,5.1)
ALB (g/L)	35.1 ± 5.9
hs-CRP (mg/L)	5.9 (1.8,10.0)
Men (%)	161 (76.7)
Age (years)	66.0 (56.0,74.0)
Hypertension (%)	176 (83.8)
Diabetes (%)	121 (57.6)
CAG (%)	210 (100)
PCI (%)	178 (84.8)
EF (%)	51.5 (42.0,62.0)
eGFR (ml/min/1.73 m^2^)	42.9 (22.5,60.8)
Hospital stay (days)	5.4 (3.1,8.3)
Killip (%)	43 (20.5)
Current smoker (%)	76 (36.2)
Left main CAD (%)	36 (17.1)
Triple vessel CAD (%)	155 (73.8)
Valvular pathologies (%)	70 (33.3)
Lab indicators	
PT (s)	13.5 (12.4,14.1)
APTT (s)	34.8 (28.2,49.7)
TT (s)	17.5 (16.7,19.4)
Hb (g/L)	116.2 ± 25.4
TG (mmol/L)	1.4 (1.1,1.9)
LDH (U/L)	291.0 (234.0,381.6)
CK-MB (U/L)	22.2 (13.1,57.3)
CK (U/L)	244.0 (94.5,629.0)
BUN (mmol/L)	12.0 (8.6,16.1)
Cr (umol/L)	170.5 (125.8,299.0)
Pro-BNP (pg/ml)	6023.0 (1352.0,16054.5)
cTNT (ng/ml)	0.9 (0.2,1.8)

NLR, neutrophil to lymphocyte ratio; PLR, platelet to lymphocyte ratio; FIB, fibrinogen; ALB, albumin; hs-CRP, high-sensitivity C-reactive protein; CAG, coronary angiography; PCI, percutaneous coronary intervention; EF, ejection fraction; eGFR, estimated glomerular filtration rate, CAD, coronary artery disease; PT, prothrombin time; APTT, activated partial thromboplastin time; TT, thrombin time; Hb, hemoglobin; TG, triglycerides; BNP, brain natriuretic peptide; cTNT, cardiac troponin T; LDH, lactate dehydrogenase; CK-MB, creatine kinase-MB; CK, creatine kinase; BUN, blood urea nitrogen; Cr, creatinine.

The cut-off values for inflammatory markers predicting MACCE were as follows: NLR 8.85, PLR 177.38, FIB 4.31 g/L, ALB 36.2 g/L, and hs-CRP 5.87 mg/L. Based on these cut-off values, patients were divided into the elevated inflammatory markers group and the control group.

### 3.2 The association between composite inflammation ratios and composite outcome

A total of 22 (10.5%) patients experienced MACCE during hospital stay, among which 4 (18.2%) patients experienced in-hospital deaths, 9 (40.9%) patients developed acute heart failure or acute exacerbation of chronic heart failure, 5 (22.7%) had malignant arrhythmias, 5 (22.7%) of fatal major bleeding, and 1 (4.5%) had acute cerebrovascular event. Logistic regression analysis was used to assess the association between composite outcome events and inflammation markers, in univariate logistic regression analysis, NLR (OR = 5.702, 95%CI: 2.269–14.328, *P* < 0.001), PLR (OR = 4.201, 95%CI: 1.572–11.227, *P* = 0.004), and hs-CRP (OR = 2.884, 95%CI: 1.152–7.217, *P* = 0.024) were associated with composite outcome events.

In the multivariate logistic regression, after confounding factors: sex, age, comorbidities (hypertension, diabetes, valvular pathologies), type of AMI, EF, smoking history, coronary situation and laboratory indicators were adjusted, NLR (OR = 14.316, 95%CI: 3.110–65.905, *P* < 0.001) and PLR (OR = 5.507, 95%CI: 1.306–22.307, *P* = 0.017) were still associated with composite outcome events (Table 2).

**Table 2:** Logistic regression analysis of inflammatory markers in CKD patients combined with AMI.

Marker	Multivariable model		
	OR	95% CI	*P* value
NLR	14.316	3.110–65.905	<0.001
PLR	5.507	1.306–22.307	0.017
FIB	0.200	0.023–1.764	0.147
ALB	0.954	0.092–9.868	0.968
hs-CRP	3.365	0.891–12.712	0.074

CKD, chronic kidney disease; AMI, acute myocardial infarction; OR, odd ratio; CI, confidence interval. Other abbreviations as in Table 1.

We further conducted point-biserial correlation analysis to evaluate the relationships between inflammatory markers and relevant covariates. As presented in Table 3, no significant associations were identified between the inflammatory markers (NLR, PLR, FIB, ALB, hs-CRP) and Pro brain natriuretic peptide (Pro-BNP) or troponin (all *P* > 0.05).

**Table 3:** Correlations of inflammatory markers with pro-BNP and cTNT.

	Pro-BNP		cTNT	
	r	*P* value	r	*P* value
NLR	0.007	0.924	−0.037	0.590
PLR	−0.014	0.842	0.031	0.654
FIB	0.076	0.273	−0.051	0.465
ALB	−0.100	0.150	0.015	0.833
hs-CRP	0.033	0.632	0.104	0.132

Abbreviations as in Tables 1, 2.

### 3.3 The association between NLR and composite outcome

**Figure 2:**
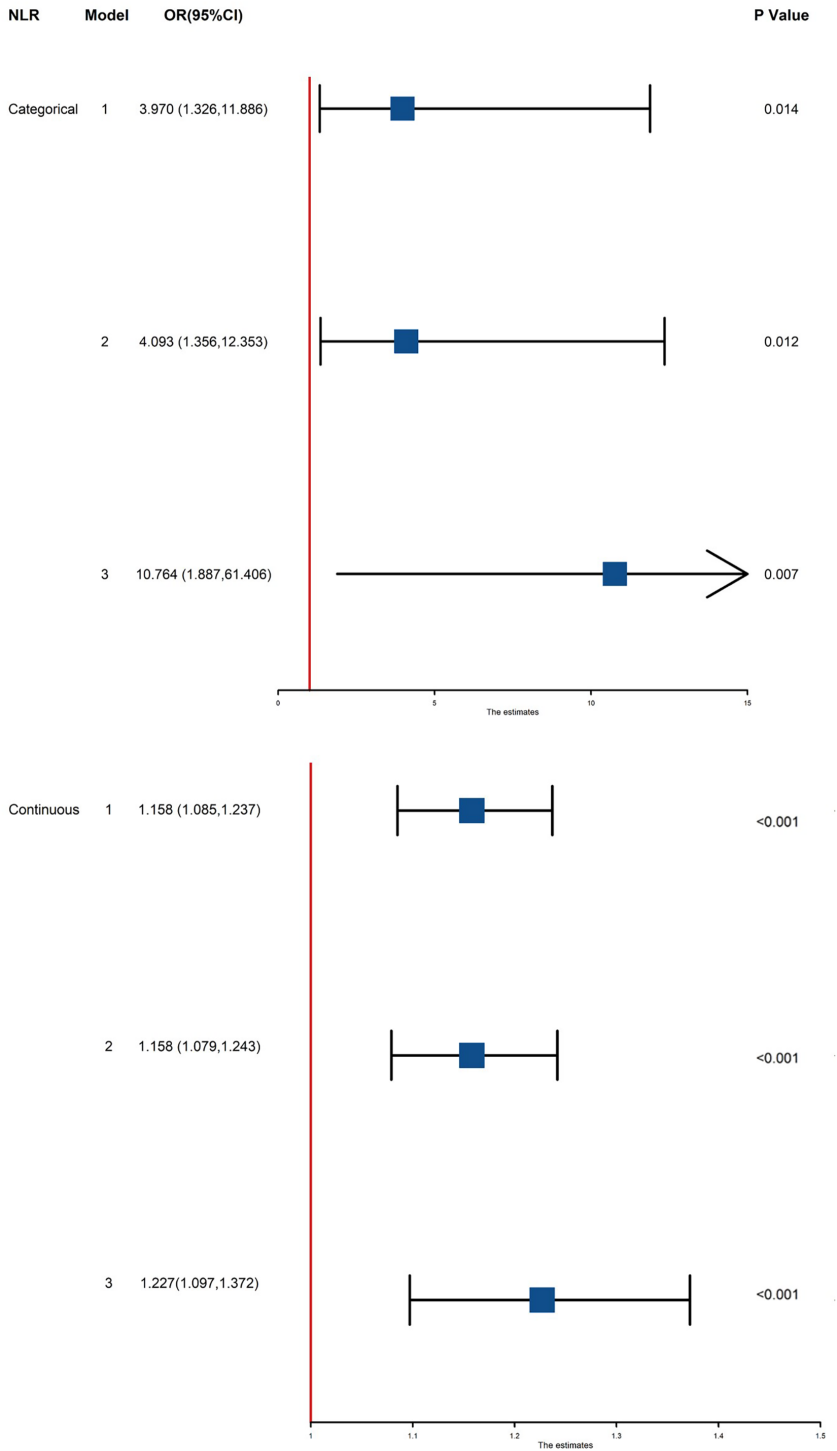
Association between NLR and MACCE in all patients. Model 1: unadjusted model; Model 2: adjusted for age and sex; Model 3: adjusted for age, sex, comorbidities (hypertension, diabetes, valvular disease, smoking history), cardiac function, coronary angiography results, and laboratory data. NLR, neutrophil-to-lymphocyte ratio; OR, odd ratio; CI, confidence interval; MACCE, major adverse cardiac and cerebrovascular events.

We included these biomarkers that showed significant effects in multivariate logistic analysis, NLR (OR = 3.970, 95%CI: 1.326–11.886, *P* = 0.014) still showed a positive correlation with a higher risk of composite outcome. After adjusting for age and sex (Model 2), this association remained significant (OR = 4.093, 95% CI: 1.356–12.353, *P* = 0.012). Additional adjustment as Model 3, the NLR still showed a positive correlation with a higher risk of the outcome (OR = 10.764, 95% CI: 1.887–61.406, *P* = 0.007) (Table 4) (Figure 2).

**Table 4:** Logistic regression analysis of related inflammatory markers in CKD patients combined with AMI.

Marker	Multivariate model		
	OR	95% CI	*P* value
NLR	10.764	1.887–61.406	0.007
PLR	1.559	0.286–8.495	0.607

Abbreviations as in Tables 1, 2.

**Figure 3:**
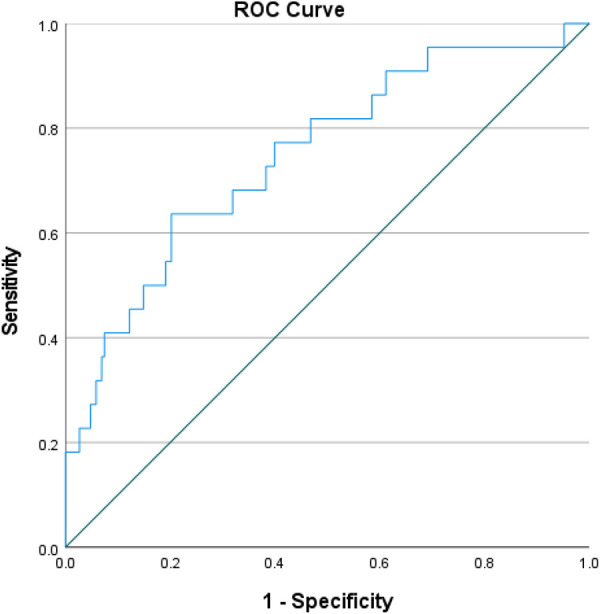
ROC curves for predicting MACCE in all patients. ROC curves for predicting MACCE plotted by the NLR in CKD patients combined with AMI. ROC, receiver operating characteristic; NLR, neutrophil-to-lymphocyte ratio; CKD, chronic kidney disease; AMI, acute myocardial infarction; MACCE, major adverse cardiac and cerebrovascular events.

**Figure 4:**
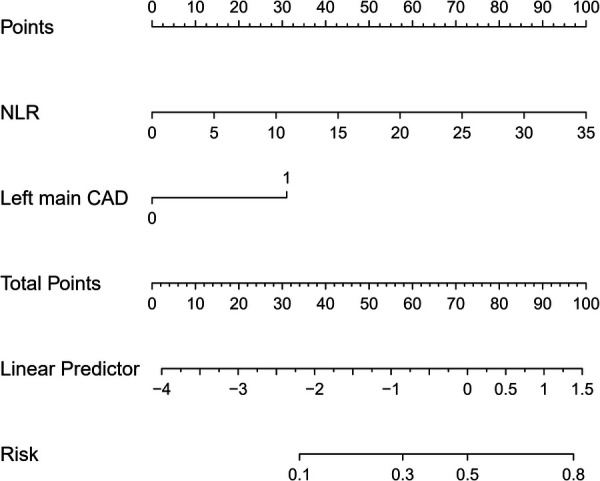
Nomogram for predicting MACCE in all patients.

As NLR was considered as a continuous variable, patients with higher levels were associated with an increased risk. After adjusting for sex, age, comorbidities, coronary situation and biochemical indicators, the group with higher levels of NLR exhibit a onefold increase in risk of the composite compared to those with lower levels (OR = 1.227, 95% CI: 1.097–1.372, *P* < 0.001). The AUC of NLR predicting MACCE in patients with AMI and CKD (0.748, 95% CI: 0.634–0.861, *P* < 0.001) (Figure 3). A nomogram was established using covariates which showed significant association with the outcome: NLR and left main coronary artery disease status (Figure 4). A point scale axis allows for each value of these variables to be assigned a score, from which the probability of the poor outcome can be estimated by summing the individual scores.

## 4 Discussion

In this study, we explored the relationship between five inflammatory biomarkers and the risk of in-hospital adverse events in CKD patients combined with AMI. The main findings of the study are: (i) among various inflammatory markers, NLR has the highest predictive value for in-hospital adverse prognosis in CKD patients with AMI; (ii) NLR is an independent risk factor for predicting in-hospital adverse prognosis in such patients; (iii) compared to lower levels of NLR, higher levels are associated with a tenfold increase in the risk of adverse prognosis.

CKD is considered as a risk factor for CVD, often independently of other common risk factors such as hypertension and diabetes (16). In a cohort study on chronic renal insufficiency, 33% of the participants (CKD stages 2–4) were found to have concurrent CVD (17). There are multiple interactive pathways between the heart and kidneys. Cardiorenal syndrome (CRS) is defined as a condition involving both heart and kidney diseases, where acute or chronic dysfunction of one organ can lead to dysfunction of the other organ (18). Patients with CKD often possess unique pathophysiological mechanisms that may play a significant role in the onset and progression of CVD. Additionally, these related mechanisms may also increase the vulnerability to plaque rupture and thrombosis, thereby contributing to the adverse prognosis in patients with concomitant AMI (19). Previous studies have found that CVD is not only the leading cause of death in the general population but also among patients with CKD (20). The reason is that patients with CKD often have high cardiovascular risk factors, dyslipidemia, oxidative stress, and inflammation, all of which contribute to the process of atherosclerosis.

Previous research has found that CKD and progressive renal dysfunction are characterized by elevated oxidative stress (21). Chronic inflammation is a key factor in promoting oxidative stress, and these two processes mutually reinforce each other (22). Inflammatory processes often have dual effects. If the process is chronic and progressive, such as CKD, it may represent a maladaptive response that promotes a series of complications. Previous studies have found that chronic inflammation is present in a significant portion of the CKD population and is increasingly associated with worsening renal function. CKD patients often exhibit high levels of inflammatory mediators, including hs-CRP, interleukin (IL)-6, and tumor necrosis factor-α, which stimulate mesangial cells, endothelial cells, and fibroblasts to produce an excessive amount of extracellular matrix. These will lead to glomerular hypertension, renal tubulointerstitial fibrosis and renal scaring (23). Systemic inflammation accompanying chronic kidney disease often leads to adverse outcomes, primarily increased mortality due to cardiovascular diseases and infectious complications. It is currently widely believed that systemic inflammation is a major driving factor for CVD in the general population, especially among CKD patients (7). Schunk et al. discovered that IL-1*α* as a central regulatory factor in inflammation for both CVD and CKD, with elevated levels being associated with an increased risk of long-term cardiovascular events (24). In CKD, various inflammatory markers may increase the production of free radicals, thereby enhancing atherosclerosis (25). Inflammation may also alter plasma protein composition and endothelial structure, thus promoting vascular disease (26). Chronic inflammation plays a crucial role in Malnutrition-Inflammation-Atherosclerosis and Calcification Syndrome (MIAC Syndrome), which is linked to adverse cardiovascular outcomes (27). Inflammation and inflammatory biomarkers play a crucial role in the pathogenesis and adverse outcomes of coronary artery disease which has been proven in previous researches (28). Inflammation is also a key factor in the onset and progression of atherosclerosis, promoting cardiovascular mortality through leukocyte adhesion and vascular endothelial infiltration (29).

Previous studies have found that after AMI, patients with CKD are associated with an increased risk of ischemic complications and bleeding, with a significant rise in both short-term and long-term mortality rates (30). Currently, an increasing number of studies are focusing on the role of inflammatory markers in patients with CKD combined with CVD. Therefore, this study selected common clinical inflammatory markers to assess their predictive value for adverse outcomes in CKD patients with concomitant AMI.

NLR is calculated by the ratio of neutrophils to lymphocytes in peripheral blood, which is more readily available compared to other inflammatory markers. Okyay, G. U. et al. found that in CKD patients, the NLR levels are higher compared to the normal population, and NLR is positively correlated with common inflammatory biomarkers such as hs-CRP and IL-6 (31). Additionally, it is thought of as a predictive indicator of cardiac mortality in CKD patients (32). Neutrophils contribute to inflammation in kidney injury through multiple biochemical mechanisms, resulting in further tissue damage. These mechanisms include the release of reactive oxygen species, myeloperoxidase, and proteolytic enzymes, all of which negatively affect kidney function (33). Yalcin Solak et al. found that CKD patients have significantly reduced lymphocyte counts, which are associated with increased inflammatory responses. Inflammation associated with CKD can increase lymphocyte apoptosis, leading to elevated risks of infections and adverse cardiovascular outcomes (34). Additionally, previous studies have found a relationship between low lymphocyte counts and poor nutritional status (35, 36). Recently, NLR has been used to assess inflammatory responses in various populations and it has also been found to be a predictive indicator of mortality in patients with various cardiovascular diseases, such as AMI (31). Increasing researches suggest that neutrophils, as part of the inflammatory response to tissue damage, may mediate plaque degeneration and instability. Elevated level of neutrophils is associated with the extent of myocardial injury and short-term prognosis in patients with AMI (37). Neutrophils release extracellular traps (NETs) containing enzymes like proteases and collagenases, making thin-cap fibroatheromas (TCFA) prone to rupture and triggering AMI. Within 24 h post-MI, necrotic myocardial tissue releases damage-associated molecular patterns (DAMPs), which bind to TLRs on neutrophils, producing a high proportion of pro-inflammatory neutrophils (N1) that release cytokines and reactive oxygen species (ROS), worsening myocardial injury. The stress response of MI activates the neuroendocrine system, raising catecholamine and glucocorticoid levels, which promote lymphocyte apoptosis and reduce their count in peripheral blood. Thus, lymphopenia is closely linked to MI severity, reflecting an intensified inflammatory response (38). Therefore, the NLR appears to be a stronger marker of inflammation than any single factor alone, consistent with our findings that NLR is an independent predictor for adverse prognosis in patients with AMI and CKD.

Additionally, this study found that high levels of PLR and FIB were associated with adverse prognosis in these patients. Kultigin Turkmen et al. found that in CKD patients, PLR levels are elevated and positively correlated with other inflammatory markers, such as tumor necrosis factor-α (39). PLR, as an inflammatory marker and a predictor of major adverse cardiovascular outcomes associated with coronary artery disease, is well-known. Previous studies have found that in CKD patients, PLR levels are positively correlated with NLR levels (40). High levels of PLR indicate excessive platelet activation and a pre-thrombotic state. Previous research has confirmed that an excess of platelets can promote inflammation and increase the risk of arteriosclerosis, thereby leading to adverse outcomes (41). Previous studies have shown that eGFR levels are negatively correlated with fibrinogen levels. Furthermore, higher fibrinogen levels are associated with atherosclerotic vascular disease and mortality in CKD patients (42). The possible mechanism is that fibrinogen participates in various coagulation processes, and acts as an auxiliary factor for platelet aggregation and an acute-phase protein (43).

Several limitations should be acknowledged. First, as this is a single-center study, the generalizability of our findings may be limited. The specific characteristics of our study population may not fully represent broader or more diverse populations, so caution is needed when applying these results to other groups. To enhance the generalizability, future multicenter studies with larger and more varied populations are needed to confirm these findings. Additionally, the retrospective design of this study restricts us to examining associations rather than causation between inflammatory markers and adverse outcomes. A prospective study design would allow for a more robust assessment of temporal relationships and causal inference, further validating the clinical utility of NLR in predicting adverse outcomes. Finally, the lack of a validation cohort limits our ability to confirm the accuracy and utility of the nomogram across other settings, which may impact its predictive capability in different populations. Despite these limitations, our study provides important insights into the role of inflammation in adverse outcomes among AMI patients with CKD, offering a foundation for future research aimed at improving risk stratification and management in this high-risk group.

## 5 Conclusion

In summary, we have confirmed that NLR is independently associated with adverse prognosis in CKD patients with AMI. High NLR can predict the risk of MACCE events in such patients. Furthermore, as a simple and accessible indicator, NLR can serve as a routine test in clinical practice.

## Data Availability

The original contributions presented in the study are included in the article/Supplementary Material, further inquiries can be directed to the corresponding authors.
